# Collectanea Medica, Consisting of Anecdotes, Facts, Extracts, Illustrations, Queries, Suggestions, &c.

**Published:** 1818-05

**Authors:** 


### COLLECTANEA MEDICA,

CONSISTING OF

### ANECDOTES, FACTS, EXTRACTS, ILLUSTRATIONS, QUERIES, SUGGESTIONS, &c.

RELATING TO THE

*History or the Art of Medicine, and the Auxiliary Sciences.*

Quicquid agunt medici, nostri farrago libelli.

*A Memoir on Stuttering:*—ψελλισμος or τραυλισμος, of the Greeks; *balbuties. hæsitatio linguæ*, of the Romans. By M. Itard, Physician to the Deaf and Dumb Institution of Paris. [Translated from the *Journal des Sciences Medicales.*]

STUTTERING is one of those impediments of the functions which, placed among our infirmities on the borders of the medical domain, has never seriously engaged the attention of physicians. The rich productions of ancient medicine are barren on this point. What Hippocrates, Aristotle, and Galen, have said on it, is hardly worth quoting, and their silence on the treatment of psellism, seems to have devoted it to absolute incurability. This is the more surprising, as this defect must have been much more distressing among a people where the art of speaking in public was intimately connected with the form of their government, and opened the road to the first honours and dignities of the state. Thus, we may remark, that those who were afflicted with this misfortune, consulted their own genius much more than the opinion of the faculty, as we learn from the manner in which Demosthenes, according to Plutarch, improved his pronunciation. Sometimes they had recourse to the gods. We read in Herodotus, that Battus, chief of a colony of Thereans, went to consult the oracle of Delphos on his stuttering, and that the advice he received was, to transport his penates under thê burning sun of Lybia.

Our therapeutic of stuttering is hardly more enlightened than that of the Pythonissa was two thousand years ago. Some observations of pathological anatomy, collected by modern authors, far from throwing any light on this affection, would rather turn us aside from its real treatment, by making us consider stuttering as occasioned by some organic lesions, such, for example, as the two accidental conduits, which, according to Sanctorius, were found in the middle of the roof of the palate, or the separation of the uvula, seen by Delius, (*Act. Nat. Curios. t.* 8.) or some defect of conformation in the *os hyoïdes*, if we may believe Hahn (*Commerc. Litter. for* 1736.) Morgagni has devoted some paragraphs of his Letters X. XI. and LI. to the etiology of stuttering, but not precisely to that which we are now considering. He only notices those embarrassments of the tongue, which are the usual remains of apoplexy, or the frequent preludes of that overwhelming disease. We are indebted to Dehaën for five or six histories of stuttering, also symptomatic, produced by congestions in the lungs, particularly by the formation of a vomica, and attended with symptoms of hemiplegia owing to the same cause. Unfortunately, these observations, related in the *Opuscules inédits* of this illustrious practitioner, are very incomplete, and badly drawn up. Still there is a fact which deserves to be remarked. In three of these patients, the expectoration of the vomica was followed by the cessation of stuttering, and of the hemiplegia.

Menjot, Fick, and Bergen, who have published dissertations on stuttering, have multiplied their divisions, and have confounded this defect of speech with other imperfections of the same organ, without indicating any rational mode of cure. Sauvages, who, in his Nosology, has copied Menjot, deserves the same reproach. It must be acknowledged, however, that he formed a true judgment of the nature of this defect, by considering it as a weakness, and by placing it consequently in the class of *dyscinesias*. It is astonishing, that he should afterwards have placed in the same class, as species of the same affection,—lallation, mogilalism, iotacism, and other faults of pronunciation, which are owing to quite different causes.

Such is the actual state of science on this subject. Let us see what my reflexions and experience have been able to add to it.

Stuttering is, as every body knows, a hesitation of the vocal organs, by which certain syllables, that require a more or less marked action of the organs of the voice and speech, are pronounced with difficulty, and with a stammering repetition of certain sounds. This defect of pronunciation is not observed in children, till having attained the age when their speech should be clear and easy, they continue to show hesitation and embarrassment in the articulation of sounds. Not but that with some attention we may perceive it, even in the first years of life, and may distinguish that imperfect articulation of sounds, those half-formed words, which mark the language of childhood from those defective repetitions of a monosyllable which constitute stuttering; but, whether from mistakes on the nature of this defect, or from hopes that it will disappear, no serious attention is paid to it till towards the seventh or eighth year, when this inconvenience, far from diminishing, becomes more apparent from the timidity of the child, and increases till beyond the age of puberty. As manhood advances, it generally diminishes remarkably, and often disappears at the approach of old age. It has sometimes been completely dispelled by an acute disorder. Timée (*Casus Medicinales*) gives the history of a stuttering child, who recovered the free use of speech about the age of eleven, in consequence of a quotidian fever. What is very remarkable, stuttering is very rare in women; and, were I to judge from my own observations, I should pronounce them totally free from it, never having seen one afflicted with it.*

To determine the cause of stuttering, we need only examine for an instant the principal phenomena which attend it. We may remark particularly, that what distinguishes this impediment of the vocal functions from all others is, that it is subject to variations of intensity depending on the moral state, which form the principal characters of nervous debilities. We may add, that among all our organs, there are none which are so completely dependent on the emotions of the mind as the organs of the voice and speech, and that, therefore, their spasmodic affections must be excited by the least agitation of the internal senses. This is precisely what happens in stuttering. Persons affected with it feel it much more in society, before a large assembly, in anger, impatience, and even in a transport of joy. In the bosom of their family, or in the calm of solitude, they speak much more freely. It diminishes also, and even disappears, if the voice is pitched on a different tone from that of conversation,—as in declamation and singing. I was consulted by a stutterer, who informed me, that he ceased to stutter when, in a large company, it grew dark, and candles were not brought in for some time, so that he could not be observed by those he was speaking to. In his youth, his parents had attempted to profit by this observation, by blindfolding him, but without success.

In the usual way, the hesitation of the tongue is particularly observed in the articulation of the consonants *K*, *T*, *G*, *L*; but

* The English Editor knew a case in a lady not less than fifty years of age. The impediment in speaking was considerable; but the lady could read with great propriety, and without difficulty.

when, from any of the reasons I have mentioned, the spasm of the vocal organ is increased, the difficulty of articulation extends to a greater number of consonants: the labial, lingual, or nasal consonants, are equally repeated; the sounds even which require only a simple emission of the voice, are, in a manner, choked in the larynx; and, the convulsive spasms, after having seized all the muscles which contribute to the voice and speech, extend to some of those of the face, and occasion very distressing distortions.

In some individuals, even the muscles of respiration, and particularly those which perform inspiration, partake at intervals of these convulsive motions, which produce a great number of aspirated sounds, that precede, or interrupt, in a disagreeable manner, the easiest words to pronounce. It was, no doubt, in allusion to this sort of stuttering, that Catullus says, in an epigram against one Arrius,—

"Chommoda* dicebat, si quando commoda vellet,
"Dicère, et hinsidias, Arrius insidias."

It is impossible not to discern in these phenomena of stuttering a spasmodic affection, and which is the result of a weakness in the moving powers of the tongue and larynx. But it is not in the marked and lengthened motions of those muscles, that this debility is perceptible. I performed some minute experiments on the tongue of a stutterer, with the design of ascertaining if its sensible motions were less free, less extensive, less powerful, than my own, but could find no difference. It is only in its delicate imperceptible motions, that this moveable organ wants the force, or rather the necessary solidity, to execute them with precision. A phenomenon may here be observed, which is more evident in some organs of motion whose functions are more easily perceptible. The muscles of the fingers, for example, may be endowed with that powerful contractility which constitutes what is commonly called a strong fist; and, at the same time, may exhibit, in those slight motions of contraction and relaxation which delicate work requires (such as writing), that hesitation and shaking which are seen in weak organs.

---

* The study of these defects of pronunciation in the works of the ancients, can alone throw some light on the manner in which they articulated sounds, or rather on the difference between their pronunciation and ours. Thus we see, by the first of these two verses, that the Romans pronounced the *h*, even when placed between the *c* and a vowel; while, in our method, it is dead to the ear, since we pronounce in the same way the first syllable of *chorus* and *coram*.

[It must here be remarked, that the French, and most other nations, are ignorant of the English sound of *ch* in *chair* and *chamber*, always giving it the pronunciation of our *sh*. The Italians soften their *c* into our *ch*, or, as other nations call it, *tch*.—Eng. Edit.]

Notwithstanding, in cases of stuttering brought on accidentally from an apoplexy, or adynamic fever, in the embarrassment which precedes some affection of the brain, all the motions of the tongue are visibly weakened. If, to be surer of it, the tongue is kept for some seconds out of the mouth, it vacillates, trembles, and yields to involuntary motions, which give it a different position from that which is attempted, and which it can only recover with hesitation. It may be remarked, in the same case, that the act of mastication and deglutition is slower and laborious. Moreover, the complete asthenic character of accidental stutterings, which all manifestly belong to paralysis, evidently show the nature of the congenital stuttering, and, I think, it cannot be doubted that their proximate cause is the same, with a few modifications; and that is, the weakness of the muscles. But is this weakness essential or symptomatic? Or, to express myself more clearly, does it reside in the muscles of speech, or is it only the consequence of some other defect? In the stuttering from childhood, I consider it to be essential; but, when it comes on suddenly, or by degrees in the course of life, it seems to depend on some affection of the brain, or some organic lesion of the instruments of the voice and speech, such as tumours at the basis of the tongue, or on the passage of the great hypoglossal nerve.—*Riviere*.

Can stuttering be cured?—I have no doubt of it; and, what I shall say directly, when speaking of the treatment of it, will help to confirm this prognostic. Many persons afflicted with this imperfection, and strongly sensible of its inconveniences, with an anxious desire to be freed from it, have succeeded by perseverance, particularly when seconded by the progress of life, which tends to diminish this defect, either by blunting that fear of displeasing which occasions timidity, or by fortifying the muscles which serve for the emission and articulation of sounds. One of the presidents of the Convention, famous for his heroical coolness and imposing eloquence in the midst of a scene of horror, was born a stutterer; but had struggled with so much success against it, as at last to overcome it. I know some other facts equally encouraging. One may hope still more, if the stuttering is in a child that has begun to speak later than usual, either from general weakness or from worms, which have often occurred, or remained long. In these cases, the epoch of puberty concurs powerfully towards the cure, by fortifying the constitution, and giving more firmness to the organs of speech. However, when the embarrassment of the tongue is considerable, the approach of puberty is insufficient to dispel it, and we must have recourse to the medical means which I shall now point out.

The proper means for curing stuttering vary according to its duration and intensity. If it be a child in whom this defect is complicated with a great volubility of the tongue, and with a confused and defective articulation in general, we must attempt to put some bounds to this immoderate use of speech, by making him spell, read with a loud voice and deliberately, often making him come back to the articulation of the syllables which he finds most difficult. Yet this method is not equal to one which I have tried twice with the greatest success. It consists in confiding the child entirely to a foreign nurse, who, only knowing the language of her own country, forces the child to learn slowly, and to give up for some years that which he had learnt too quick. To this plan, I once added that of keeping the organs of speech in complete repose and silence for a whole year; as, from continual hesitations and involuntary fatiguing repetitions, they appeared to me to be prematurely charged with a function beyond their strength.

These means produce but little change, when the individual has passed the age of adolescence. At this epoch the practice of declamation would be very useful. He should begin by that, which, being the most remote from the tone of conversation, requires more slowness and precision in the motions of the tongue, a louder voice, and longer kept up,—as in tragedy or preaching. From that he will proceed to more familiar declamation; and, at length, to reading comedies in prose; performing these exercises as much as possible before a numerous society.

In all cases, it is of much importance, in order to carry on the cure with method, to distinguish what is fundamental and permanent in this defect, from what arises through anxiety and apprehension on speaking in society, or in public; for, if the stuttering was always the same, (which is exceedingly rare,) or but little susceptible of variation, it would be sufficient to exercise frequently the organs of speech in the articulation of difficult sounds, to remove the impediment. These practices, so advantageous in the case we are speaking of, are not useless in any, and I cannot recommend them too strongly. But, to proceed with method, one should thoroughly know the theory of vocal sounds, both simple and articulate. For this the works of Wallis, Ammann, and the Abbé Lépée, should be carefully studied, and which, on that account, I shall not discuss in this memoir. To the precepts of these writers, I shall only add one important particular, which none of them have mentioned; which is, that it is not sufficient, in order to familiarise the tongue with the articulation of sounds, to study the mechanism of that articulation, and subject it to frequent repetitions, but that we must exercise ourselves with these articulate sounds in all their possible combinations. A syllable which is fairly pronounced, if preceded by another which leaves the tongue in a favourable situation for overcoming the difficulty, will not be so easy, if it follows some other which has not that advantage, or if it forms the beginning of a word or phrase; just as one consonant will occasion more frequent or stronger stuttering, if connected with such and such a vowel, than with another; which is commonly observed in the letter *c*, which stutterers pronounce with more difficulty when it is followed by an *a*, than when it precedes an *o*.

But, when the stuttering is susceptible of increase, and the embarrassment extends to a great number of syllables, and even simple sounds, it is not sufficient to render the articulation of sounds easier and more correct; we must then endeavour, by the application of mechanical means to the organs of speech, to augment their strength in overcoming the spasmodic susceptibility. We must do for the muscles, destined to the formation of speech, the same as is practised for the muscles of locomotion, to which steadiness and suppleness are given in proportion as they are employed in various or fatiguing exercises, such as dancing and fencing. From this comparison, and in order to obtain an analogous exercise from the muscles of the tongue and lips, I render their motions more difficult and laborious by obstacles placed in the mouth; and, notwithstanding the great uneasiness they occasion, one must still talk, shout, sing, and whistle, with them frequently. At first, it is impossible; but, after some days, and by repeated efforts, the muscles, overcoming the impediment, recover their motions, and, what is more, with greater steadiness. To produce this desired effect, I make use of a very simple instrument.—It is a sort of little fork, made of platinum or gold, which rises from the concave centre of a flat and bent stem composed of the same metal, and applied with its convex side to the concavity of the alveolary arcade of the inferior jaw-bone. The fork, which is supported by this metallic arch, is an inch long, more or less, placed in a horizontal situation opposite the *frænum:* it receives that membranous check in its bifurcation, while its two branches, terminated each by a flat button about the size of a bean, rest on the inferior side of the tongue in the returning angle which it forms by its union with the inferior side of the mouth.

This instrument is scarcely fixed in its place, when, as I have said above, a confused and embarrassed voice is heard, very analogous to that which characterises the erosion or congenital division of the veil of the palate; but, what is remarkable, quite free from stuttering. The most difficult syllables are painfully articulated, but by no means repeated; and this favourable change still continues, even when the organs of speech, accustomed to the instrument, have recovered the freedom of their motions, and can pronounce sounds clearly articulate. However, if this mechanical support be too soon removed, the stuttering comes on as before; it must, therefore, be kept on a long time, and, when it is necessary to remove it, as for eating or sleeping, silence must be rigorously observed. I cannot precisely say how long it is necessary, being able to cite only two instances of a cure by this method. One was a young man, twenty years of age; he kept this sort of *bit* in his mouth for a year and a half, and found it so little troublesome at last, that, during some months, he did not even take it out to eat. The perseverance with which he bore this apparatus arose from a motive which, at that age, will make one undertake and support any thing,—the hope of pleasing a young woman with whom he was violently in love, and whose coldness he attributed merely to his unfortunate stuttering. His complaint, indeed, was very bad, and subjected him to convulsive exacerbations of the muscles of the mouth, nose, and eye-lids, which made speaking both painful to himself and unpleasant to the company. His success was complete; but I never heard whether he obtained the object of his wishes.

The second object of my observation was a boy eleven years old, who could not feel the same motives for perseverance. He was very impatient with the instrument, and took it out of his mouth whenever he was not watched. Notwithstanding which, when he was brought to me after eight months, the stuttering was considerably diminished; and, though I have heard nothing more of him, I am almost certain that a few months more would have completed a cure. At the moment I am writing this, a young man, passed thirty, who one day or other will take his hereditary seat in the Chamber of Peers, has subjected himself to the same treatment with a constancy and strength of mind inspired by the most noble motives. A sensible improvement, within a few weeks, gives me reason to think, that I shall not have undertaken his case in vain. The instrument which I have had made for this young man is much more perfect than those which I previously employed. I am indebted for it to the skilful hand of Pernet, the dentist. Moveable branches, shorter or longer, more or less divergent, allow the pressure on the muscles of the tongue to be increased, and the resting points to be varied. I have seconded the effects of these mechanical means by tonic gargles, made with an alcoholic tincture of bark, cantharides, and other stimuli.

There are few cases of stuttering in which this last method might not be used with advantage. I must, however, except that sort of stuttering (very rare indeed) in which the organs of the voice, much more than those of speech, appear to be the seat of that spasmodic hesitation which suddenly suspends the formation of words. It is perceptible from the sounds appearing, as it were, to be stopped in the larynx, and it becomes evident, if, on attempting to pronounce with some haste a long series of vowels, the difficulty still recurs. In this case, most advantage will be derived from the practice of vocal music, and particularly from that exercise of the voice which consists in stringing sounds together. These means may be seconded by some local tonics. Moxa, on the sides of the larynx, and the *os hyoïdes*, might have the happiest effect. I recommended it once in a stuttering of this kind; but, the patient taking alarm, it was not employed. In prescribing this mode of excitation, I was guided by the good effects which I have often observed from it in chronical aphonia, essential dumbness, and other lesions of the voice and speech, which will form the subject of another memoir.

[The great prevalence of the hooping-cough induces us to insert the following. Though the suggestions are not new in this metropolis, yet we are informed, that, in remote parts of the country, the continued use of emetics, and the change of air in the early period of the complaint, are still continued.]

### *On the Hooping-cough;* by William Gamage, jun. M.D.

The hooping, or chin, cough seems to have been less understood than any other disease of so common occurrence. It has been handed over to empiricism by almost common consent. Influenced by the aspect of a majority of its cases, physicians have been in the habit of viewing it as a mild disease, and worthy little consideration. Its mischiefs have been attributed to other causes, and when it has terminated in the death of its subject, some other disease has taken the odium. I had been accustomed to view it in this light, till the autumn of 1815, when I met with several cases, so severe in their symptoms, and, at the same time, so unequivocal in their character, as to put it beyond a question, that the hooping-cough, unaccompanied with any other affection, could be a formidable disease. The death of one interesting boy was so evidently the effect of this disease alone, that no room was left for deception or even doubt. A detail of his symptoms is given in Case I.

It is but recently, that any attempt has been made to ascertain the true character and seat of this disease; at least, the only source which could lead to any certain knowledge on these subjects has been neglected;—I mean the examination after death of those who have been its victims. Dr. Watt, of Glasgow, has lately obliged the profession and society with a very able work on this subject; and this appears to be the first attempt at a philosophical investigation of the disease. He relates some cases which had been previously communicated to the public by Dr. Lettsom, and which evinced the true spirit of inquiry; but these had attracted little attention till this notice. Dr. Watt suffered most cruelly in his own family, by the loss of two of his children by this disease. He magnanimously permitted their bodies to be examined after death. The morbid phenomena, discovered in these instances, induced him to pursue the investigation. His views as to the nature of the affection were changed; and every new case served to strengthen his conviction, that the disease belonged to the class of inflammatory complaints. He was, therefore, led to adopt a mode of practice different from those in common estimation. This consisted principally in a more liberal use of the lancet; and, from this practice, he experienced the happiest consequences. I shall not pretend at this time to give an account of the contents of this work. It is a most valuable acquisition to medical literature, and deserves the attention of every physician. I shall be happy, if the following histories add any thing to the information contained in this volume.

Case I.—W. J. a stout boy, two years old, had the hoopingcough, which commenced sometime in the latter part of October. The cough, I was informed, became violent soon after he began to hoop. I saw him on the 10th of November. The symptoms most apparent at this time, were the severity of the fits of coughing, and some hardness of the pulse. He had a cathartic and a vomit of the tartrite of antimony, which was to be repeated occasionally. On the 27th, he had taken three effective emetics and syrup of garlic and squills in liberal quantities, but his symptoms had become progressively worse. The cathartic and the emetic repeated; nauseating doses of the tartrite of antimony and squills were frequently repeated. He was put into a warm bath, and had a bath of warm vinegar constantly applied to his chest. 19th.—No amendment, but paroxysms of fever occurred twice every twenty-four hours; the breathing was hurried by slight exertion. It was particularly noticed, that, when the child was awake, the breathing was short and quick, but, in sleep, it seemed perfectly easy. I have remarked the same occurrence since. It seems to be a good mark, by which we may distinguish the inflammation of the mucous membrane from pneumonia. In the latter, the breathing is at all times hurried, and performed with difficulty. The pulse was quick and small; the cough-fits severe and frequent, particularly during the night. I took from a vein on the back of the hand about three ounces of blood, and applied a large blistering plaster to the chest.

20th.—Seemed a little better; but the pulse remained unchanged. Had a cathartic of the sub. M. Hydrarg.

21st.—Symptoms less favourable. The frequent and long fits of coughing, the febrile paroxysms, the hurried breathing, and the quick and small pulse, evinced the disease to be unabated. He was again bled to the amount of four ounces. The blood was quite thin, coagulated slowly, and exhibited a buffy coat. The sub. M. Hydrarg. was given, one grain every four hours.

23d.—Some mitigation of the symptoms was evidently obtained by the bleeding; but they soon regained their former severity. A repetition of the bleeding was objected to. An emetic, therefore, and the warm bath were again tried.

24th.—Getting worse; pulse intermits. Blister renewed upon the chest. The cough was particularly distressing during the night. Opiates of the assafœtida gave no relief.

No advantage was obtained over the symptoms. He died on the 27th. In the two last days the cough abated, and he was disposed to sleep continually. The skin had been in a hot and moist state.

*Dissection.*—The body was examined eighteen hours after death. The lungs were of their natural colour, and appeared to be sound. They exhibited externally no mark of inflammation; had no unnatural hardness in their substance, as when death takes place from pneumonia. No tubercles, no pus, could be discovered; but, when cut into, a frothy and apparently semi-purulent fluid issued from the air-cells.

The trachea and bronchiæ were next examined. A large quantity of semi-purulent mucus occupied these parts. When this was removed, the mucous membrane appeared of quite a florid colour. This was more intense in the bronchiæ, and quite conspicuous in the membranous extremities of their branches. The vessels of this membrane seemed to be injected with blood, and a bright redness, of a radiated appearance, was observed in different spots. The semipurulent matter was quite adhesive in the bronchiæ: the smaller branches were filled with it, and it could be pressed in abundance from the air cells. These appeared to me to be strong marks of inflammation.

The pericardium and heart were found in a natural state. The abdominal viscera were examined, but no morbid appearances discovered. The stomach had not the least mark of inflammation.

The result of this case, and the morbid phenomena which it exhibited after death, convinced me of the propriety of bleeding in this complaint; and that, in cases of so much severity, no other remedy ought to be trusted. This was the first instance of a fatal termination of this affection, unaccompanied with any other that I had witnessed; and, though sensible that bleeding was a proper remedy, I was not aware of the extent to which it was necessary to use it,—and, therefore, contended less earnestly against the common prejudice than I should have done, had I had my present conviction of its necessity. I did bleed, but not so early in the case, nor so copiously, as its urgency required. I had not then seen that excellent work of Dr. Watt on this disease, but was feeling my way to truth unassisted by the light of others.

I did not witness another bad case of this complaint till January, 1817. It was frequent, especially in the autumn and December of 1816; but it was mild, and seldom required medical aid. In the latter part of January, and in February, the weather became severely cold, and many bad cases of hooping-cough occurred in my practice. I experienced the happiest effects from bleeding. I used it in most cases when the cough was severe, and always with certain alleviation. I have on record ten cases, whose symptoms were rendered mild by a single bleeding, when emetics, and the empirical remedies in common use, had given no relief. In some of these cases, the fits of coughing or kinks were so violent as to cause blood to gush from the nose and ears during the paroxysms. In others, there was an expectoration of blood. The subjects were from four to ten years old, and the quantity of blood taken from each was from four to eight ounces. A detail of these cases would be uninteresting; but, in the following, the symptoms were more complicated, or more unyielding.

Case II.—C. H. G. a delicate girl, aged seven months, had the hooping-cough. It progressed mildly for about four weeks: the fits of coughing then became more severe and distressing. She seemed at first relieved by occasional antimonial emetics; but, in a short time, other bad symptoms appeared. She had febrile paroxysms; restless nights; foul bowels; water evacuated only once in twenty-four hours; pulse quick and small; breathing hurried, when awake. In sleep, the breathing was perfectly easy. Bleeding seemed to be the only remedy that promised any success in this case, and this could not be effected without great difficulty. It was impossible even to feel a vein on the arm or hand, and the external jugular was so buried in fat, as to be quite indistinct. I succeeded, however, in getting from this vein about an ounce and a half of blood. This seemed to give some relief, but it was of short duration. On the second day after, I made another attempt, with about the same success; but the relief seemed greater, and was more lasting. All the symptoms were mitigated except the cough, and this occurred less frequently. Her situation seemed flattering for four or five days, when, after a violent kink, she had a convulsive fit. An emetic was immediately given, the bowels opened by injections, and a large blister applied to the chest. The usual medicines for moderating the cough had been given perseveringly: among these was the syrup of garlic, which appears to be a useful remedy for the cough when there is little inflammation present. A solution of assafœtida was now added to the remedies, and the warm bath was used, but all in vain: the fits again recurred in the twenty-four hours. I attempted again to bleed, and succeeded in getting two ounces of blood from the same vein. I would have taken more, but the child could not be induced to cry, or make such exertion as to cause the blood to flow. It seemed to be improved by the bleeding, and was a little roused from the stupor in which the last fits had left it: twenty-four hours elapsed before they again recurred; but, after this, they did not cease till death relieved the little sufferer.

*Dissection.*—The body was examined eighteen hours after death, in the presence of Dr. Hayes and Dr. Jackson. The thorax being laid open, the lungs collapsed as much as usual. They were a little more florid than in ordinary cases, particularly the middle lobe of the right lung, which had a vermilion colour; but there was no hardness or other mark of pleuritic or pneumonic inflammation. When their substance was cut into, the usual frothy fluid oozed out. The trachea was opened posteriorly, the lungs being removed from the body. The first thing noticed was an adhesive mucus, of a yellowish colour, in considerable quantity in the trachea and bronchiæ. This being wiped away, the mucous membrane appeared more flushed than is natural. In the lower part of the trachea, in the spaces between the cartilaginous rings, the colour was a bright red. In the bronchiæ there was a more general floridness, and this was particularly bright at the origin of their branches, and it could be traced into the membranous parts of these tubes. No fluid of any degree of purulency was perceived in this case; nor had the stomach and intestines the least mark of inflammation: the coats of the stomach were remarkably white.

The inflammation of the mucous membrane, in this case, was not sufficient, I conceive, to cause death. In fact, the hooping-cough was evidently relieved previous to the commencement of the convulsive fits; and it probably was not the cause of death, otherwise than it might be the means of inducing the fits. It is not improbable, that the brain of so tender an infant should suffer by being so frequently injected and crowded with blood, as it must necessarily have been by the violence of the coughing-fits. The veins of the scalp of this infant seemed ready to burst whenever it had a paroxysm of coughing. It is to be lamented that circumstances prevented the examination of the head, as it is not improbable that there would have been found the proximate cause of the fits and of death. Had a greater quantity of blood been obtained, previous to the commencement of the fits, I am disposed to think the result of the case might have been favourable.

Case III.—February 6th, 1817.—S. B. a girl, aged three years, had had the hooping-cough in a mild way, the mother supposed, about five weeks. On the 2d, its fits of coughing were observed to be more violent than usual: they daily increased in severity. She became feverish, and breathed with difficulty; she had taken emetics and cathartics, and had the warm bath, without any permanently good effect. She was asleep when I saw her, and seemed to breathe quite easy: pulse quick and rather hard, but not so small as in most similar cases—it gave to the finger a peculiar grating sensation. When the child awoke, her breathing was hurried and laborious. Her mother had noticed this difference in the breathing in her sleeping and waking hours. The face was swollen and flushed; skin hot, and the tongue coated. Bleeding seemed to me to be the only appropriate remedy, and I proceeded to take, from a vein on the back of the hand, four ounces of blood. A bath of warm vinegar was kept constantly applied to the chest.

7th.—Symptoms very little improved: she had two severe febrile paroxysms during the last twenty-four hours. The bleeding was repeated, and over four ounces of blood was taken from a similar vein as yesterday. Some common medicines were given to relieve the cough.

8th.—She was not so much better as to be trusted without additional bleeding. Four ounces of blood were again taken.

9th.—In all respects better. The mother, an intelligent woman, remarked, that she had never witnessed such evident relief as was afforded her child by the last bleeding. She had a gentle cathartic.

10th.—Symptoms not quite so favourable as yesterday: had some heat, and a pulse too quick: breathing somewhat difficult. Took away four ounces of blood.

After this there was no difficulty in the management of the case. The child entirely recovered, and is now healthy and vigorous.

The inflammation in this case appeared to me to be principally confined to the mucous membrane.

The symptoms which distinguish the inflammation of this membrane from that of common pneumonia, or pleurisy, seem to be—first, the pulse is not so small in that of the first as in that of the latter diseases; secondly, the patient has no fixed pain; thirdly, in pneumonia and pleurisy, the respiration, with children at least, is always short and accompanied with a sort of grunt; and it is for the most part the same, whether the patient be asleep or awake; but in the inflammation of the mucous membrane, the inspirations are deeper, and not usually attended with the grunt; and, in sleep, it is comparatively easy.

Case IV.—S. C. G. a healthy girl, aged three years, had the hooping-cough so mildly, that she continued at school, and seemed very little incommoded by it. At the end of the fifth or sixth week she seemed more unwell, and the cough became much more violent than it had been. She complained much of her head, and was observed to hold it with both hands, when the kinks occurred. Any considerable motion, or looking steadily at an object, caused dizziness and pain in the head. She had an emetic and a cathartic; but the cough continued so severe, that blood was forced from her nose and ears during the kinks. The cathartic was repeated on two succeeding days, and obtained some relief to the head, but the pulse had become very unpleasant: it was full, remarkably slow, about sixty-five in a minute, and intermittent. Serious apprehensions were entertained as to what might follow. Bleeding seemed to be the proper remedy; but to do this from the jugular vein was extremely abhorrent to the parents, and it was impossible to see or feel a vein either on the arm or the back of the hand. The morning after, this state of the pulse was particularly noticed: she appeared more ill, and about noon was suddenly prostrated in a state resembling a fit. I saw her immediately: she lay in a state of stupor; face flushed; skin hot; pulse very rapid and small. Bleeding could be no longer delayed: the jugular vein was opened, and between four and five ounces of blood extracted. Almost immediate relief was obtained. The present symptoms after some hours subsided; but the pulse again became slow and intermittent, but not so crowded as before. It had another peculiar trait,—a strong grating sensation under the finger. Her head was relieved, but the countenance retained its look of distress and anxiety. The character of the cough had entirely changed; it was small, and no longer attended with a hoop. These symptoms continued much the same for some days. She took the sub. M. Hydr. in doses of one grain every six hours; had cathartics frequently repeated, and occasionally an emetic, when demanded by some peculiar symptoms. As soon as the affection of the head had subsided in a tolerable degree, the cough became more troublesome. The hoop, however, did not return, and the child slowly recovered perfect health.

I have heard, from medical gentlemen, of several instances of affections of the head during the hooping-cough. In one case, the patient seemed to die with symptoms of hydrocephalus, and water was found in the ventricles of the brain after death. The patient of another gentleman was apprehended to have hydrocephalus, but it recovered. This patient was supposed to have been benefited by blisters alternately applied to the head and chest, and by the digitalis, which seemed to produce great evacuations of water by the kidneys. The cessation of the kinks was observed, in the last case, when the head began to be affected; and their return as the head recovered.

The remarkably slow and intermittent pulse, mentioned in the above case, was noticed in another child of the same family. He was a stout boy, about two years old; he had the complaint mildly, and, except this state of the pulse, had no very severe symptom. The slowness of the pulse was pretty constantly present, but the intermission only occasionally observed.

Case V.—April 1, 1817, I was called to S. P. a girl aged one year nearly. She was in a most distressing situation with the hooping-cough. Her kinks were frequent and violent; breathing hurried and laborious; skin hot; tongue thickly coated; pulse quick and small. She had had the complaint, by account, between five and six weeks; mild at first, but, for the week past, had been getting worse. She was a fat and plump child previous to the attack of the disease; but she had become quite thin, and her skin hung loosely about her. She had been treated liberally with emetics and many empirical remedies, but had derived no apparent advantage from them. The case was of little promise, but bleeding had not been tried. I took two ounces of blood from a vein on the back of the hand, and directed her to be put into the warm bath, and, after this, the vinegar bath to be kept to the chest. 2d. —Symptoms a little relieved: she lost two ounces more of blood. 3d.—She was in many respects better, had less fever, better nights, and kinks not quite so frequent; but they were still violent, and proceeded almost to strangulation: breathing also still bad. I took from the jugular vein three ounces of blood in a full stream: she was a little faint. The orifice in the vein was covered with a court-plaster. Some time after, in a fit of coughing, the orifice again opened, and it was judged that about two table-spoonfuls of blood were lost.

The child after this recovered rapidly, without farther medical aid.

In this case, the inflammation, though principally confined to the mucous membrane, had affected in some degree, I conceive, the substance of the lungs. This is not an uncommon occurrence. Pneumonia often supervenes upon the hooping-cough, and, in every such instance, it arises much more probably from the spreading of the inflammation from the mucous to the cellular membrane of the lungs, than from the influence of any external cause, to which it is generally attributed. When this takes place to any great extent, the situation of the patient is extremely hazardous, and, unless promptly relieved, almost desperate. Hooping-cough has terminated fatally in this way more frequently than in any other. In this state of the disease, bleeding seems to be the only remedy that merits any confidence.

The following cases are examples of the disease in this form.

Case VI.—February 14th, Abraham A. a lad aged six years, had the hooping-cough mildly about five weeks, when the kinks became severe; and, continuing so about a week, he was suddenly attacked, six hours before I saw him, which was in the evening, with difficulty of breathing, and an obtuse pain in the side. Skin hot; pulse rapid and small. Took from him immediately six ounces of blood, and applied a bath of warm vinegar to his chest.

15th.—Morning. Symptoms very little improved: bled him to the amount of four ounces, and directed a large blister to the chest.

16th.—Heat and pain diminished: breathing performed with more ease, but the pulse still quick and small. Four ounces more of blood were taken.

After this he was convalescent. The pneumonic symptoms disappeared, and with them the hooping-cough. He had a slight cough, but it soon left him.

Case VII.—A. A. Creech, eight years old, began to be affected with the hooping-cough about the middle of January. It progressed mildly for the first five weeks. He occasionally expectorated a little blood, and bled at the nose; but had no other unpleasant symptom, except, almost from the first, a quick and sharp pulse. In the sixth week, the fits of coughing became more severe. February 22d, I saw him, in the morning, in a strong paroxysm, and his pulse was worse than it had been. It was thought prudent to bleed him, and four ounces of blood were extracted from the arm: it was quite florid, and coagulated slowly. I was called to him again in the evening. He had some pain in the chest, laborious breathing, hot skin, and a pulse as rapid as could be counted. These symptoms, without any exposure, commenced about seven hours after the bleeding in the morning. A repetition of the bleeding seemed to me the only chance of relief; six ounces of blood were, therefore, immediately taken from him: it coagulated sooner than in the morning, and had a firm buff. A large blister was applied to the chest.

23d.—Breathing easier; pain less; skin cool; but the pulse very little improved. Bled him to the amount of four ounces.

The pulse remained quick and small, but other bad symptoms disappeared, and not a vestige of the hooping cough remained. He had slight cough, which gradually subsided; his appetite returned, and he was able to be up and with his play-mates; but he made no progress: pulse did not improve; and, after a few days, he evidently grew worse. His flesh diminished; food rejected; and he had frequent febrile flushes: but he had no pain, slept well, and breathed with perfect ease. His situation, however, was bad. An emetic was administered, small doses of the sub-muriat. hydr. often repeated, and a vesicatory applied over the place of the former pain. He obtained little advantage from these means, but continued in much the same state till March 18th, when he was suddenly taken with strangulation, and, after a minute, coughed, and brought up (according to the report of his attendant) a roundish substance, of the bigness of a large walnut, consisting of a soft substance, which he said looked like matter; and of small round bodies, which were harder. This was followed by considerable fresh blood. In about twenty-four hours after, a similar substance was coughed up, of less bulk, and accompanied with less blood. They were, most unfortunately, not preserved for my inspection. From the description, and from what I have seen in some dissections, I am disposed to think that they were clusters of small tubercles, which had been insulated by the suppuration of the surrounding substance, and, being separated, made their way into a neighbouring bronchial tube by ulceration, and were thence ejected. Immediately after this occurrence the lad began to mend: appetite good, and the food retained. He has since become stout and active.

Case VIII.—S. H. aged twenty months, had the hooping-cough in January, 1817. When the disease had continued about four weeks, she began to suffer from the violence of the cough, and frequent febrile paroxysms. She was bled two days in succession, to the amount of two ounces each time. The symptoms were relieved, but not removed. Fifteen days after this, I was again called to her: she was very ill with pneumonia, and had been so for two or three days. Three ounces of blood were taken from her. I was not permitted to repeat the operation: blistering, emetics, &c. were tried in vain: she died in about seven days from the attack.

The body was examined twenty-four hours after death. The lungs were generally of a dark colour, and crowded with blood: the right lung was extremely diseased, and some parts of it as hard as liver; the middle lobe, particularly, was very hard, and nearly one half of it of a yellowish white colour. This portion consisted of a collection of distinct roundish bodies, connected together by a membrane; they were, for the most part, of a firm consistence, and resembled diseased bronchial glands. Well formed pus was found in the centre of some of them; in others, a substance like cheese-curd. This nucleus was partly enclosed by the natural substance of the lungs, and its boundaries well defined. It appeared to me to be a collection of tubercles, which, as they increased in size, had destroyed the intermediate substance of the lungs. I could not believe it to be a recent disease, and it seemed that the child had a severe pneumonia when it was about a year old. Pus was found in every other part of this lobe, lodged in sinuses, many of which communicated with the cavity, which contained the above nucleus. In the other lobes of this lung much pus was found, and numerous small tubercles.

The left lung seemed little diseased: some tubercles, however, and some pus, were found in its inferior portion.

The bronchial glands were generally diseased. They were observed in clusters, and very much enlarged; some of them as hard almost as bone, others of the consistence of firm marrow.

The mucous membrane of the trachea exhibited spots of a dark red colour. These were more numerous in the bronchiæ, and, in the smaller branches, this colour was quite general; and as much so in those which belonged to the sound as to the diseased lobes. The bronchiæ were filled with a semi-purulent matter, of a reddish tinge: the pus seen in the lungs had not found access into them. The mucous membrane of these tubes was particularly noticed to be thickened, and to have lost its natural glossy smoothness.

CASE IX.—S. G. E. aged six years, had had the hooping-cough about six weeks. The cough had been severe, but, as he had not been otherwise unwell, it was not regarded. February 4th, he began to droop, and his kinks were more frequent and violent. On the 6th, he was suddenly taken with great difficulty of breathing, and a febrile paroxysm. I saw him in the evening: he lay in a state of stupor; breathed laboriously, and with great rapidity; face flushed and swollen; skin hot; pulse very small and quick. I took from him immediately six ounces of blood, and applied a large vesicatory to his chest. He had been previously well evacuated by a cathartic.

7th.—Symptoms not materially improved: respiration and pulse much as yesterday. Took from him three ounces of blood in the morning, and three in the evening.

9th.—Respiration performed with a little more ease; pulse not improved; coughed more than for two days past, and expectorated florid blood. Urine scanty, high coloured, and loaded with mucus. The most active epispastics had no effect on the skin. Two ounces of blood were taken from him in the morning, and two in the evening.

9th, 10th, and 11th.—The symptoms did not improve so much as to afford any encouragement for ultimate success. The respiration continued quick and short; pulse quick and small; florid blood still expectorated. He was bled to the amount of two ounces each day, and he was slightly blistered. After the bleeding on the 11th, there seemed to be a ray of hope: he breathed with tolerable ease, the pulse became frequent and fuller, and he had a little appetite. This appearance of amendment lasted till the 13th. On the 14th he was not so well, and continued failing, till (the 16th) he died.

*Dissection.*—The body examined sixteen hours after death. The lungs were of a light colour at their upper, and dark at their under, surface, from the subsidence of the blood. They had, to the sight, the appearance of being sound; but, when pressed between the fingers, numerous small tubercles could be felt in almost every part of them: these were hard, and varied in size from a small shot to a large pea: they were most numerous in the middle lobe of the right lung. This lobe was the most diseased, and, when cut into, exhibited pus in numerous insulated globules. Pus was discovered in other parts of the lungs, but not so abundantly as in this. The only portion that retained this hardness, which is a common effect of pneumonic inflammation, was a part of the inferior lobe of the left lung.

The mucous coat of the trachea and bronchiæ was, in this case, not in the least red; but it was evidently thickened, opaque, and deprived of its natural glossy smoothness, particularly in the smaller branches of the bronchiæ.

The inflammation in this case appeared to have been almost entirely removed, even from the mucous membrane. But the injury which that membrane had sustained, and the general diseased state of the lungs, seemed to me to have been the immediate causes of death. The pus observed in the substance of the lungs must have been the effect of recent inflammation; and I am disposed to have the same opinion as to the existence of the tubercles,—they were very numerous and small. The bronchial glands were not diseased.

The information to be derived from these cases, relates,—first, to the nature and seat of the disease; and, secondly, to the proper means of cure.

With respect to its seat, it seems to be well ascertained that the principal affection is in the mucous membrane of the trachea and bronchiæ. In all dissections after death by this disease, this membrane has been discovered to be in a morbid state; and, though, in some instances, other parts have been found to have suffered, to the affection of no other could the peculiar symptoms of hooping-cough be so reasonably assigned. Cases 1st and 2d of this paper were genuine examples of this disease: in these cases, this membrane was the only part in which the effects of previous morbid action appeared.

As to its nature, it is probable that we shall at present remain in the dark with regard to what constitutes its essence. All that we are able to discover are the effects of inflammation; and to this cause we must attribute all that is either distressing or dangerous in the disease. From the general aspect of the symptoms, and from the morbid phenomena disclosed by dissection, we infer that inflammation is uniformly present, and that it makes an essential part of the disease. In this respect it does not differ from other specific diseases, all of which are accompanied with a greater or less degree of inflammation, and their severity and hazard are in proportion to its activity and extensiveness. Whether this inflammation has any thing specific in its character, the morbid changes do not enable us to determine; and, as it regards the treatment, it is not material, as the same means will control it in either case.

From this view of the complaint, the treatment should consist in the use of such means as are the most effectual in reducing inflammation. Among these, blood-letting, without question, holds the first place; but, besides its fitness for this purpose, experience of its utility sanctions its employment. It is not, however, necessary to bleed in every case of hooping-cough; but I am persuaded that, even in common cases, there would be infinitely less suffering during the progress of the disease, were this remedy employed; and there is really no harm in taking a little blood from a child, though it has so formidable a look. At the worst, it is much less injurious, in my estimation, than repeated emetics: but, with the present weight of prejudice against it, few probably will be disposed to recommend it, except in severe cases. In these, in order to ensure its greatest advantage, it must be employed without delay, and with perseverance. I would bleed as soon as the cough became severe; and at this time one liberal bleeding would in most cases prevent mischief, and render the disease mild in its progress. But, upon the accession of the febrile paroxysms, and the hurried respiration, which indicate a great degree of inflammation in the mucous membrane; or when the inflammation has extended to the substance of the lungs, and thus produced, in combination with those of the hooping-cough, symptoms of pneumonia, this remedy becomes indispensable to the safety of the patient. Under these circumstances, we must not be satisfied with a single bleeding, but it must be repeated in liberal quantities, till the symptoms are under control, or we are convinced that amendment is beyond the power of the art. A timid use of this remedy is to be deprecated. It often happens that a first bleeding effects no apparent improvement in the state of the disease, when a second or a third would completely accomplish the object. If abandoned after a single trial, the patient loses the best means of relief; the remedy is brought into discredit, and has laid to its charge many of the evils, really the effects of the disease. It is equally bad policy to delay this remedy till every other has been tried. If employed in the last stage of the complaint, it will probably do no good, and will thus get into disrepute, if not with the physician, certainly with the lookers-on. I am persuaded that, in most instances of its failure to relieve this disease, (and I might, perhaps, add other acute diseases,) it is to be attributed to its being resorted to too late, or used too sparingly.

Next to blood-letting, antimonial emetics afford us the most efficient means of cure. But, if there be any affection of the head, cathartics, particularly of calomel, seem to have the happier effect.

Blisters are, probably, useful remedies, but they have appeared to me to do less good in inflammations of the mucous membrane, than in pneumonia or pleurisy. An efficient use of the lancet will render their employment rarely necessary in either form of the disease.

[Our own opinion, confirmed by long observation, is, that the early treatment should be, invariably, cathartics with blood-letting, if the inflammatory symptoms require it. These all well attended to, the patient will rarely require any other emetic than what is caused by the act of coughing. As soon as the disease becomes chronic, the treatment must be varied according to the symptoms, but change of air is always desirable.—English Edit.]

